# Zeolitic intralayer microchannels of magadiite, a natural layered silicate, to boost green organic synthesis†
†Electronic supplementary information (ESI) available: Experimental details and additional data (Table S1 and Fig. S1–S11). See DOI: 10.1039/c8sc03712d


**DOI:** 10.1039/c8sc03712d

**Published:** 2018-11-02

**Authors:** Yusuke Ide, Satoshi Tominaka, Hiroyuki Kono, Rahul Ram, Akihiko Machida, Nao Tsunoji

**Affiliations:** a International Center for Materials Nanoarchitectonics (MANA) , National Institute for Materials Science , 1-1 Namiki , Tsukuba , Ibaraki 305-0044 , Japan . Email: IDE.Yusuke@nims.go.jp ; Email: TOMINAKA.Satoshi@nims.go.jp; b Department of Earth Sciences , Waseda University , 1-6-1 Nishiwaseda , Shinjuku-ku , Tokyo 165-8050 , Japan; c Center for Education , CSIR-Central Electrochemical Research Institute , Karaikudi , Tamil Nadu , India 630006; d Synchrotron Radiation Research Center , National Institutes for Quantum and Radiological Science and Technology , 1-1-1, Kouto, Sayo-cho , Sayo-gun , Hyogo 679-5148 , Japan; e Graduate School of Engineering , Department of Applied Chemistry , Hiroshima University , 1-4-1 Kagamiyama , Higashi-Hiroshima 739-8527 , Japan

## Abstract

Despite the considerable attention given to the applications of magadiite in previous research, the properties of this natural layered silicate have remained mysterious due to the lack of crystal structure information. On the other hand, no one has doubted the intercalation capability *between* the layers. Here we succeed in determining the structure of magadiite using X-ray pair distribution functions and synchrotron powder diffractometry. We discover unexpected zeolitic microchannels *within* the layers. We describe efficient synthesis of 100% pure benzoic acid from toluene by using magadiite as an additive in a TiO_2_ photocatalytic system oxidizing toluene. Based on the uncovered structure of magadiite, we clarify the mechanism of this unique photocatalytic system: the microchannels of magadiite not only separate/accommodate the desired partially oxidized product formed on TiO_2_ but also prevent the accumulation of the overoxidized products on the TiO_2_ surface that deactivates the photocatalytic activity.

## Abstract 2

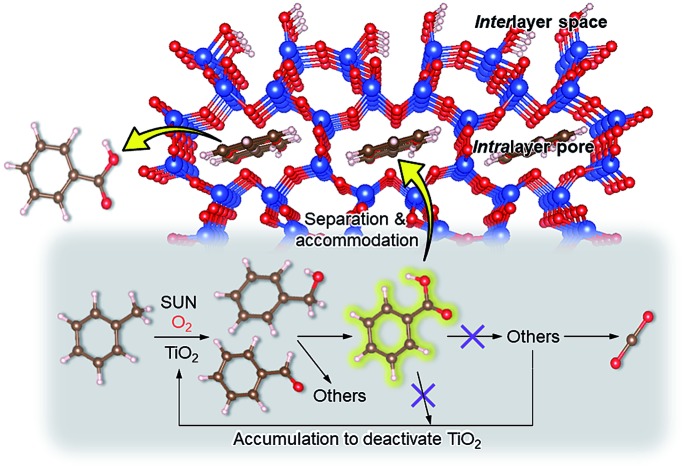
We discovered unexpected intralayer microchannels of magadiite, a natural layered silicate, and used it as additive in a TiO_2_ photocatalytic system oxidizing toluene to realize efficient and selective synthesis of benzoic acid not achieved by conventional photocatalysts.

## Introduction

Development of microporous materials such as metal–organic frameworks and zeolites, with unique and better properties, is crucially important, while yet challenging, from scientific and practical viewpoints.^1^ Owing to their ubiquity in nature and/or low cost, combined with high thermal and chemical stability, layered silicates such as layered clay minerals have a strong historical background in the field of microporous materials (or scaffolds for designing microporous materials).^2^–^4^ Magadiite is a naturally occurring layered silicate, which was found at Lake Magadi, Kenya in 1967 (ref. 5) and is nowadays one of the most frequently studied layered silicates for various uses, for instance, as an adsorbent and catalyst.^6^–^15^ In terms of large-scale production, magadiite is attractive because it can be facilely prepared by hydrothermal reactions. However, its properties are still mysterious because the crystal structure had not been solved yet due to its crystal habit forming fine, lamellar crystallites, which tend to have stacking disorders (namely, low crystallinity). Here we succeed in determining the structure of magadiite for the first time and report on the presence of unexpected zeolitic micropores within the layers.

We also report on the use of magadiite to boost green fine chemical synthesis. Oxidation of organic chemicals to high-value products through environmentally friendly routes is crucially important and remains a challenge for the sustainable production of fine chemicals for use in the chemical, pharmaceutical and agricultural industries. One such reaction is oxidation of toluene to partially oxidized products like benzoic acid, which is one of the most highly sought partially oxidized products from toluene. Commercially, benzoic acid is mainly produced *via* cobalt-catalyzed reaction of toluene with O_2_ at 140–190 °C under a pressure of 10 atm, where the conversion is limited to <15% to retain a high selectivity.^16^ Development of efficient and selective catalysts for the reaction is key and thus has been addressed for decades,^17^ but these conventional processes have issues such as the usage of expensive reagents and materials as well as strong conditions.

As an alternative to the conventional ones, environmentally friendly processes for synthesizing fine chemicals have been investigated. Solid photocatalysts like TiO_2_ can produce benzoic acid from toluene in the presence of O_2_ with solar energy even under ambient conditions. This research using photocatalysts for benzoic acid production is promising but is in its infancy; for example, benzoic acid is not produced selectively due to side reactions such as overoxidation, and the oxidation rate of toluene is slow.^18^–^24^ It is clear that both selectivity and yield have to be improved for the successful exploitation of these photocatalytic systems. In this study, 100% pure benzoic acid is recovered in a significantly high yield by using magadiite as an additive capable of separating/accommodating the product in a TiO_2_ photocatalytic system oxidizing toluene.

## Results and discussion

We revisited the structural analysis of magadiite, the natural form containing sodium ions, named “Na-magadiite”, and its protonated form, “H-magadiite”, and finally succeeded in solving their atomic structures through X-ray pair distribution function (PDF) analysis with the help of composition analysis data and other characterization data such as X-ray diffraction (XRD) and solid-state ^1^H and ^29^Si NMR data (Table S1 and Fig. S1–S7†) (details are available in the Experimental section and ESI†). As written in detail in the methodology section, we analyzed the atomic arrangement of magadiite by solving the local-to-medium-range structure by PDF analysis, determining the unit cell from the XRD pattern and then determining the crystal structure by the real-space method using the network structure obtained by the PDF analysis.

**Fig. 1:**
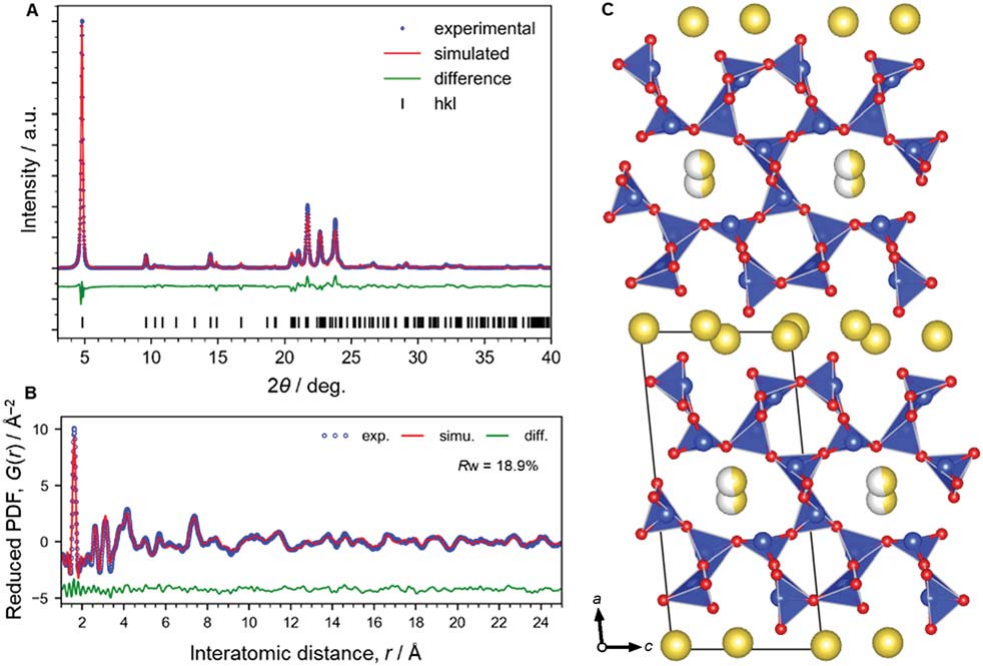
Structural analysis of Na-magadiite. (A) Synchrotron XRD pattern analyzed by the Rietveld method. (B) X-ray pair distribution function (PDF) analyzed by curve fitting. (C) Structural model obtained by the PDF analysis. This is basically consistent with the structural model obtained by the XRD (details are available in the ESI†). Colour coding: blue = Si, red = O, yellow = Na.

The XRD analysis (Fig. 1A) shows that Na-magadiite adopts a triclinic structure in the *P*1 space group (Na_1.44_Si_10_O_22_·3H_2_O);^7^–^11^ *a* = 15.75(4) Å, *b* = 3.930(3) Å, *c* = 7.365(5) Å, *α* = 96.382(13)°, *β* = 95.92(4)°, *γ* = 96.18(8)°, and w*R* = 8.69% (details are available in the ESI†). The crystal structure has large isotropic atomic displacement parameters (*U*_iso_) of >0.2 Å^2^ (available in the ESI†), indicating the presence of short-range chemical orders as often found in silicates. Note that the X-ray scattering intensities from Na ions and H_2_O molecules are similar; thus we modelled them using Na atoms. The PDF analysis (Fig. 1B) for revealing local-to-medium-range orders indicates a structure consistent with the crystal structure well (*a* = 15.60(3) Å, *b* = 3.837(7) Å, *c* = 7.344(14) Å, *α* = 93.76(19)°, *β* = 95.42(17)°, *γ* = 95.21(18)°, refined by PDF in the range 0.5–25 Å, *R*_w_ = 18.9%). These structures obtained by the XRD and PDF (Fig. 1C) analyses are consistent with each other (Fig. S1 and S2†). The structure consists of ∼1.2 nm thick silicate layers (the *d* spacing is 1.5 nm, which includes the interlayer space), along which micropores filled with Na ions coordinated by water molecules (and OH anions as charge-compensating anions) exist as zeolites.

We describe the chemical formula for the unit cell as H_*n*_Si_10_O_22_·1.44[Na(H_2_O)_4.0_] (*n* = 1.7), taking the amounts of (i) Si and Na determined by inductively coupled plasma optical emission spectroscopy (ICP-OES, Table S1†) and (ii) H_2_O molecules determined by thermogravimetric analysis (TGA, Fig. S3†) into consideration (details are available in the ESI†). The amount of silanol groups, SiOH (*n* number), was determined from the TGA data, and it probably underestimated the amount due to possible presence of remaining silanol groups (note that the value expected from the charge balance and NMR results (Fig. S4†) is 2.56).

**Fig. 2:**
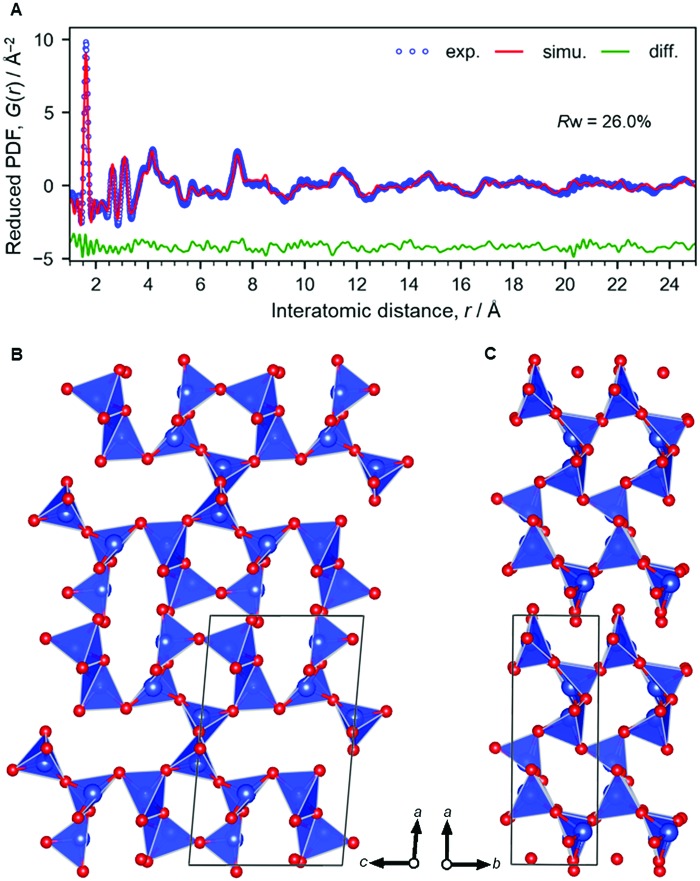
Structural analysis of H-magadiite. (A) X-ray PDF analyzed by the curve fitting. (B and C) Structure model. Colour coding: blue = Si, red = O.

By acid treatment, the Na ions were removed and elliptical channels remained in the structure of H-magadiite as clearly found by the PDF analysis (Fig. 2, Si_10_O_22_·*n*H_2_O (*n* < 0.3); *a* = 12.06(2) Å, *b* = 4.134(8) Å, *c* = 7.393(15) Å, *β* = 94.80(17)°, refined by PDF fitting in the range 0.5–25 Å, *R*_w_ = 26.0%). Note that we analyzed the Na-magadiite structure and found that the space group of the framework only (without Na and water molecules) could have additional symmetry. Then, we used the monoclinic cell in the *P*2_1_ space group for the PDF analysis of H-magadiite. The modest fitting quality is probably due to the presence of stacking disorders found in the XRD pattern (Fig. S8†) or partial hydration of the structure, but it is good enough to confirm the framework structure, which is in good agreement with the silicate framework in the Na-magadiite structure (Fig. 1, S1 and S2†). We confirmed that, like octosilicate, another widely studied layered silicate,^25^,^26^ Na cations in Na-magadiite were removed with structure retention. Likewise, the chemical formula of H-magadiite is H_3.6_Si_10_O_22_·*n*H_2_O (*n* < 0.3).

**Fig. 3:**
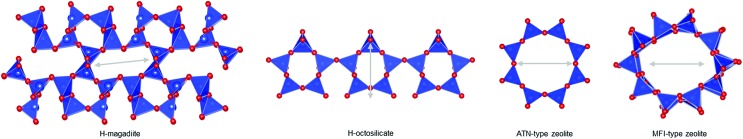
Characteristics of the H-magadiite framework. Single layers in H-magadiite and H-octosilicate, and 8-membered and 10-membered rings in ATN-type zeolite and MFI-type zeolite (silicalite-1), respectively. The length of grey arrows is identical (6 Å).

The silicate layer of H-magadiite (∼1.2 nm) is thicker than that of other protonated layered silicates like octosilicate as suggested previously^11^ because of the presence of microchannels defined by the eight-membered rings of SiO_4_ tetrahedral networks (Fig. 3). Compared with octosilicate, which is composed of four-, five- and six-membered rings, and other many layered silicates, the eight-membered-ring channels are unique. The channels are aligned along the layers and accessible to a certain kind of molecule and ion as the channels are formed by Na ions coordinated by water molecules. Note that X-ray PDF analysis suggests the presence of the Na(H_2_O)_*n*_ complex in the channels but the coordination number and long-range orders are not clear.

**Fig. 4:**
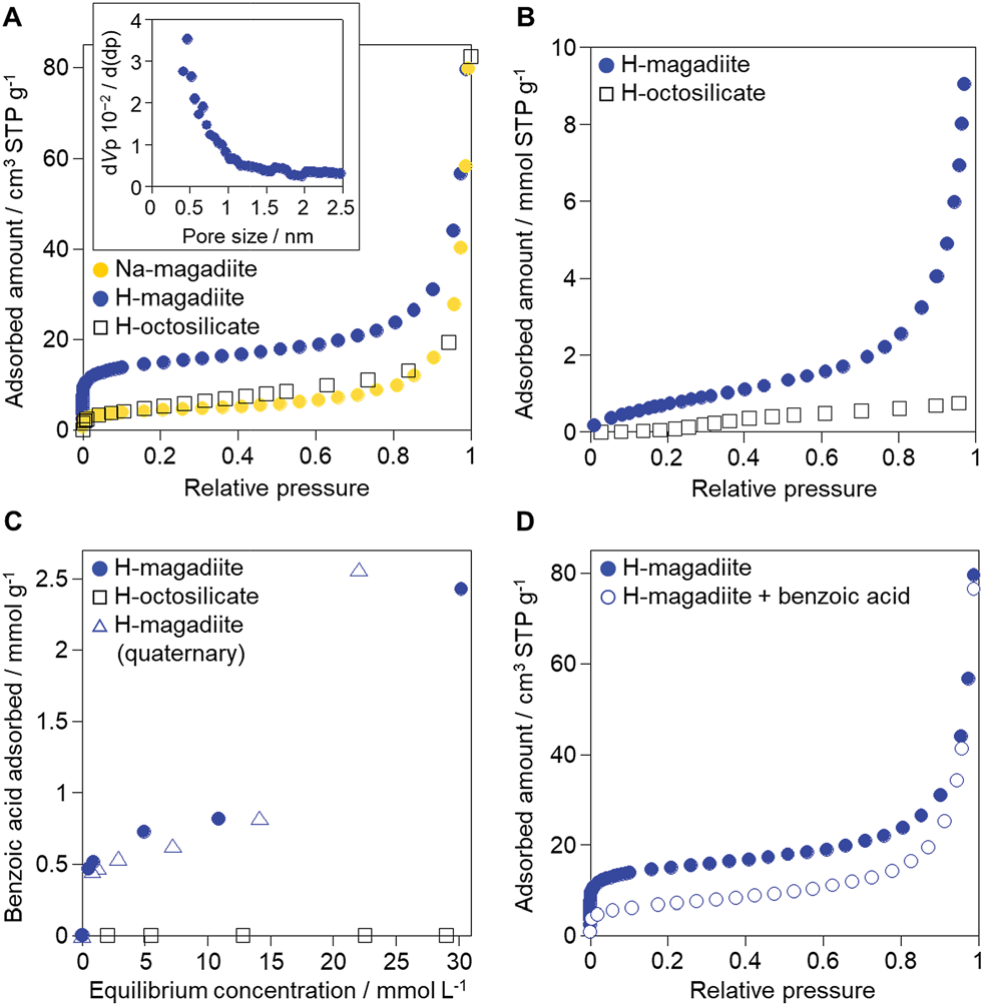
Adsorption properties of H-magadiite. (A) N_2_ adsorption isotherms of Na-magadiite, H-magadiite and H-octosilicate. The inset shows the pore size distribution of H-magadiite by the Horvath–Kawazoe method applied to the N_2_ adsorption isotherm. N_2_ adsorption/desorption isotherms and BJH pore size distribution of Na-magadiite and H-magadiite are shown in Fig. S9.† (B) Water vapor adsorption isotherms of H-magadiite and H-octosilicate. (C) Adsorption isotherms of benzoic acid on H-magadiite and H-octosilicate from acetonitrile solution. The adsorption isotherm of benzoic acid on H-magadiite from a mixed solution of acetonitrile containing toluene, benzyl alcohol, benzaldehyde and benzoic acid is also shown. (D) N_2_ adsorption isotherms of H-magadiite before and after adsorption of benzoic acid.

Interestingly, the experimental data indeed indicate the porosity of the H-magadiite. For example, the N_2_ adsorption isotherm (Fig. 4A) shows that N_2_ molecules adsorbed onto H-magadiite at low pressure, indicating the presence of micropores. The BET area is 54 m^2^ g^–1^, which is significantly higher than that of Na-magadiite (15 m^2^ g^–1^). The micropore volume (by *t*-plot) is calculated to be 0.022 cm^3^ g^–1^ (0.001 cm^3^ g^–1^ for Na-magadiite), which is close to the total volume of the calculated voids associated with the microchannels (0.022 cm^3^ g^–1^ for the space 0.2 Å away from the surface; 0.041 cm^3^ g^–1^ for the space 0.0 Å away from the surface, Fig. S10.† Note that the voids were calculated for all the space silicate atoms do not occupy, and thus must contain too narrow space for N_2_ molecules to access. The pore size is estimated to be *ca.* 0.5 nm (Fig. 4A inset), which is in good agreement with crystallographic calculations (this will be discussed in detail later). In contrast, very little N_2_ adsorption was observed for octosilicate (protonated octosilicate named H-octosilicate), which has only smaller rings within the layers.^25^,^26^ These facts indicate permanent porosity of the framework of H-magadiite. Such N_2_ adsorption into the eight-membered-ring channels is reasonable because a synthetic layered silicate, AMH-3, which has three-dimensionally connected channels defined by eight-membered rings within the layers, also exhibits similar N_2_ adsorption behavior.^27^ Unlike AMH-3, the framework of H-magadiite is not rigidly connected, which probably causes the flexibility of the framework (it is reasonable to consider this on the basis of the structures of Na-magadiite before and after acid treatment, Fig. 1C and 2B).

In addition to the unique micropores, the interlayer space is also important for understanding magadiite's properties. The interlayer space is formed by the silicate surface having silanol groups like those of other layered silicates,^2^–^4^ but the surface of the H-magadiite layers is covered with a large amount of silanol groups (2 per 30.6 Å^2^ of the *bc* plane), twice as many as those on H-octosilicate (2 per 54.46 Å^2^). The interlayer hydrogen bonding in H-magadiite was reported to be quite strong based on solid-state ^1^H NMR experiments,^11^ which probably results from the higher silanol density than in H-octosilicate. Water vapor adsorption, which is often used to estimate the hydrophilicity of microporous surfaces depending on the surface silanol groups,^13^ confirmed that H-magadiite is more hydrophilic than H-octosilicate (Fig. 4B). The shape of the isotherm and the capacity of water vapor for H-magadiite is in good agreement with reported data,^28^ in which H-magadiite can adsorb water molecules *via* interactions with the surface silanol groups to slightly expand the interlayer space.

The role of H-magadiite in the selective synthesis (recovery) of benzoic acid is attributable to the presence of micropores. Fig. 4C shows the adsorption isotherms of benzoic acid on H-magadiite and H-octosilicate from acetonitrile solution. H-magadiite exhibited L-type adsorption according to the Giles classification,^29^ indicating strong adsorbent–adsorbate interactions, while H-octosilicate scarcely adsorbed benzoic acid. The shape of the isotherm obtained in the presence or absence of toluene, benzyl alcohol and benzaldehyde, other partially oxidized products from toluene,^16^–^24^ was quite similar. These results indicate that H-magadiite specifically adsorbs benzoic acid while it scarcely adsorbs other aromatic compounds (indeed, the adsorption of toluene, benzyl alcohol, benzaldehyde and acetonitrile on H-magadiite was not detected as described below).

The structural information on H-magadiite enables us to interpret the mechanism of its specific adsorption of benzoic acid. Eight-membered-ring channels in zeolites are well known to be too small to adsorb benzene rings (Fig. 3). One may wonder if the benzoic acid adsorption reshapes the channels to adsorb the molecules likewise, but the PDF of the sample with benzoic acid adsorption is identical to that of plain H-magadiite (Fig. S8†), meaning that such reformation is not obvious. The XRD patterns reveal that the adsorption changes the relative intensities while retaining the diffraction angles; in particular, the peak at the lowest angle of 5.62° (*d* = ∼13.2 Å) was increased by the adsorption, meaning that benzoic acid exists in the structure, not on the external surface, and the structure expands only by ∼1 Å along the stacking direction. These facts strongly suggest that benzoic acid molecules exist in the microchannels. The adsorption of benzoic acid in H-magadiite is saturated at an equilibrium concentration of around 10 mM, and the amount is estimated to be 0.8 mmol g^–1^ (Fig. 4C), which corresponds to 0.5 molecules per unit cell. Comparing the lengths of the benzoic acid molecule (∼8 Å) and channels in a unit cell (∼4.1 Å), the adsorption amount suggests full filling of the pores.

The size of the channels in H-magadiite was analyzed by crystallographic calculations (details are shown in Fig. S10†). The channels are solvent-accessible as found by the adsorption experiments, and their cross-sections are *ca.* 5.5 Å wide and *ca.* 2.1 Å high. This width is slightly larger than the width of aromatic rings of ∼4.9 Å based on covalent bonds (H–H distance, 4.3 Å, and atomic radius of a H atom, 0.3 Å), but the molecules may be large enough to experience van der Waals repulsive force from silicate walls (the van der Waals radius of a H atom is 1.1–1.2 Å; the molecular size is ∼6.5 Å based on van der Waals radii^30^). Thus, the aromatic rings have almost the same size as that of the channels and may experience weak repulsive force, which may result in a slight expansion of the structure as found by the XRD analysis. This in turn suggests the importance of hydrogen bonding, which induces attractive force between molecules and silicate (ROH···OSi). In fact, non-planar and/or aprotic aromatic molecules, toluene, benzyl alcohol and benzaldehyde cannot enter the microchannels (Fig. 4C). Benzoic acid, a planar protic molecule, can be adsorbed selectively. Though the pore size is tight for benzoic acid molecules, it is not surprising that they diffuse inside if the framework is flexible as reported for coordination polymers.^31^

To confirm the adsorption of benzoic acid in the intralayer channels, N_2_ adsorption on benzoic acid-adsorbed H-magadiite was investigated (sample at equilibrium concentration of *ca.* 10 mM in Fig. 4C). As shown in Fig. 4D, the amount of N_2_ molecules adsorbed onto H-magadiite at low pressure dramatically reduced upon benzoic acid uptake. This is explained by the fact that benzoic acid molecules inside the microchannels prevent the entrance of N_2_ molecules.

**Fig. 5:**
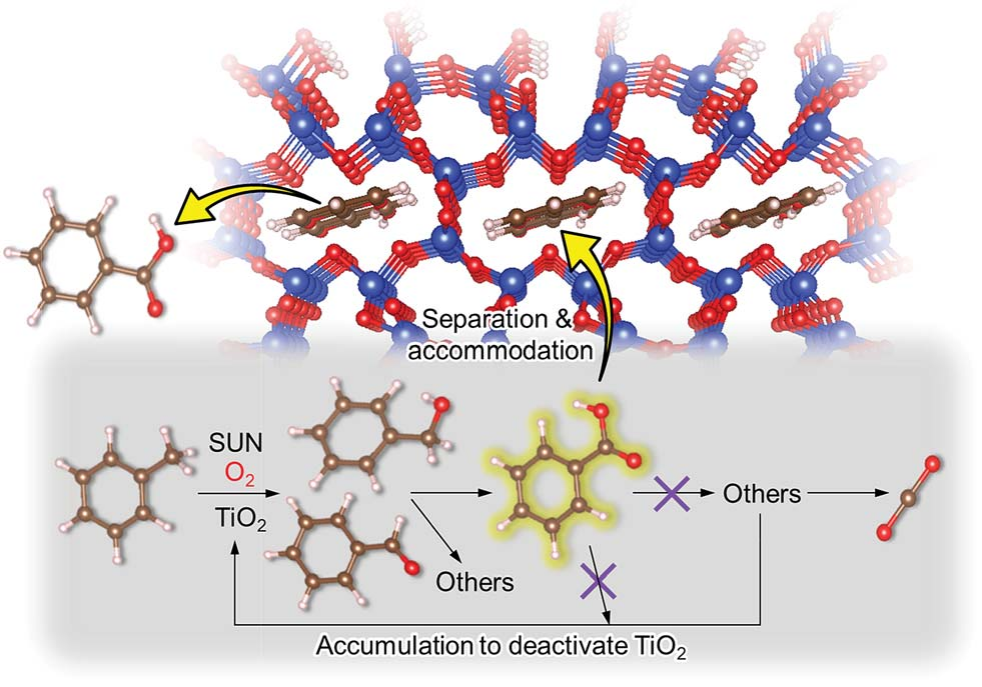
Schematic representation of the mechanism of the enhanced partial oxidation of toluene on the TiO_2_ photocatalyst with the aid of the H-magadiite additive. H-magadiite separated benzoic acid; thus, the accumulation of benzoic acid and its reacted products on the TiO_2_ surface, which deactivates TiO_2_, is inhibited.

On the basis of this understanding of H-magadiite, we adjusted the experimental conditions for the photocatalytic oxidation of toluene by TiO_2_ in the presence of the H-magadiite additive, which can separate/accommodate benzoic acid. Especially, in contrast to our preliminary study for synthesizing phenol from benzene using water as a solvent of benzene,^32^ the use of non-aqueous solvent (acetonitrile) is key to increasing the amount of the added toluene and then improving the yield of the product (Table 1). When TiO_2_ alone was used, only trace amounts of benzoic acid, benzaldehyde and benzoic acid, and the completely oxidized product (CO_2_) were detected in the supernatant and head-space (<1% selectivity) gas even at a toluene conversion of 30%. This indicates the formation of a variety of products as reported in the literature^18^–^24^ and reveals again the difficulty in selectively synthesizing partially oxidized products of toluene *via* photocatalysis. On the other hand, when H-magadiite was present under identical conditions, 100% pure benzoic acid was eluted from the recovered H-magadiite with high yield (recovery of 22%). Benzoic acid was hardly detected in the supernatant during the course of the photocatalytic toluene oxidation (Fig. S11†). Given that TiO_2_ is known to photo-oxidize toluene mainly through benzyl alcohol and benzaldehyde to benzoic acid,^17^,^22^ H-magadiite adsorbed the formed benzoic acid selectively, effectively and rapidly, preventing the further reaction of benzoic acid such as its coupling with benzyl alcohol and overoxidation as expected (Fig. 5).

**Table 1:** Photocatalytic oxidation of toluene on TiO_2_ with and without adsorbents

Adsorbent	In the supernatant and headspace gas						In the eluate^*a*^			
	Toluene conversion^*b*^ (%)	Product selectivity^*c*^ (%)					Recovery^*e*^ (%)	Purity^*f*^ (%)	Recovery^*g*^ (%)	Purity^*f*^ (%)
		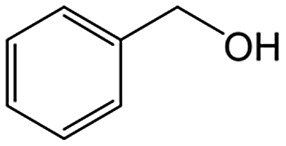	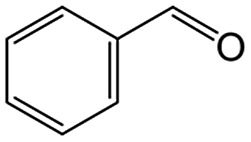	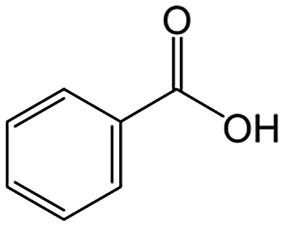	Others^*d*^	CO_2_	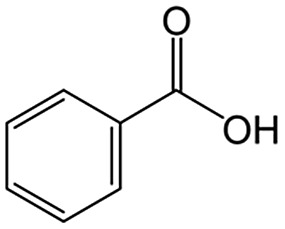		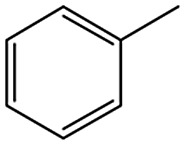	
None	30	0.2	0.4	n.d.	99	0.3	n.d.	—	n.d.	—
H-magadiite	89	n.d.	0.1	n.d.	75	0.1	22	100	n.d.	—
H-octosilicate	29	n.d.	n.d.	n.d.	99	0.3	n.d.	—	n.d.	—
Silicalite-1	9	n.d.	n.d.	0.003	98	2.1	n.d.	—	0.2	100

^*a*^Solution obtained after washing the recovered solids with aqueous ethanol.

^*b*^[Reacted toluene]/[added toluene] × 100.

^*c*^[Product]/[reacted toluene] × 100. For CO_2_ selectivity, 1/7[product]/[reacted toluene] × 100.

^*d*^Calculated as 100 – [selectivity for other products].

^*e*^[Benzoic acid]/[added toluene] × 100.

^*f*^Based on GC.

^*g*^[Toluene]/[added toluene] × 100.

Remarkably, H-magadiite addition allowed not only the recovery of benzoic acid in 100% purity but significantly improved the toluene oxidation rate (conversion was improved from 30 to 89% as shown in Table 1). Generally, TiO_2_ is deactivated due to the accumulation of oxidized products, including those derived from benzoic acid, on active sites during the photocatalytic oxidation of toluene.^21^ In the present case, H-magadiite adsorbs benzoic acid to keep the TiO_2_ surface active (Fig. 5). We thus realized the photocatalytic partial oxidation of toluene with both high selectivity (purity) and high yield (recovery) that could not be attained even by using advanced photocatalysts.^23^,^24^

To confirm the above scenario, other additives were tested. With H-octosilicate, the photocatalytic activity of TiO_2_ scarcely changed (Table 1). This was predicted from the adsorption properties of H-octosilicate (Fig. 4). On the other hand, with silicalite-1, toluene conversion was significantly lowered and only toluene was detected in the eluate (Table 1). Silicalite-1 has relatively large 10-membered ring channels (Fig. 3); thus, the silicalite-1 additive adsorbed toluene effectively on the hydrophobic channels to decrease the toluene oxidation rate on TiO_2_. This result shows the merit of H-magadiite as an additive of TiO_2_ photocatalytic systems oxidizing aromatic compounds for organic synthesis.

## Conclusions

We revealed that magadiite has micropores aligned in the extremely thin layers as well as dense, strong hydrogen bonds formed in the interlayer space in H-magadiite. These structural features of magadiite are expected to create advanced applications of magadiite as we demonstrated, and, of course, we should revisit/sophisticate the conventional materials design of magadiite and other layered silicates; for instance, structure information enables us to design zeolites and unique inorganic–organic hybrids having properties that cannot be found in conventional microporous materials more rationally through condensation and silylation of the silanol groups of magadiite.^12^,^13^ The dense silanol groups and the information on their arrangements may also enable us to design single-site catalysts of transition metals (*e.g.*, Ti and V) grafted on silanol groups.^4^,^33^

## Experimental section

### Preparation of materials

Na-magadiite was purchased from Nippon Chemical Industrial Co. Ltd., and was used as received. The preparation of H-magadiite and H-octosilicate was carried out based on previous reports.^26^,^34^ Full characterization data of Na-magadiite and H-magadiite are provided in the ESI.† P25 was supplied from Nippon Aerosil. Pt-loaded P25 (Pt/TiO_2_ = 0.2 wt%)^35^ and silicalite-1 (ref. 36) were prepared according to previous reports. The as-prepared silicalite-1 was calcined at 600 °C for 6 h.

### X-ray PDF measurements

X-ray total scattering data for obtaining pair distribution functions (PDFs) were collected on a Rigaku Rapid-S curved imaging plate detector with Ag Kα radiation (*λ* = 0.556 Å) for screening structure models initially. The samples were sealed in Cole-Parmer polyimide capillaries (inner diameter: 1.0 mm). These corrected intensities were normalized by the Faber–Ziman type scattering form factors calculated using atomic scattering factors to obtain structure functions, *S*(*Q*), using the MaterialsPDF program.^37^ The *S*(*Q*) (*Q*_max_ = 21.0 Å^–1^) was treated with a revised Lorch function (Δ = 1.00),^38^ and then converted into reduced PDF, *G*(*r*), where *r* is the interatomic distance.

High-resolution PDF data were obtained using synchrotron irradiation at BL22XU (*λ* = 0.1774 Å) and BL08W (*λ* = 0.1076 Å) of SPring-8 with a PerkinElmer flat panel detector (XRD1621). The collected image data were converted into a scattering intensity profile using the PIXIA program.^37^ The former results in PDFs with reasonably good spatial resolution, *Q*_max_ = 25.5 Å^–1^, and also good angular resolution enabling analysis of the long-range region in real space. The latter results in PDFs with high spatial resolution, *Q*_max_ = 33.0 Å^–1^, but with little angular resolution.

### Details of structure analysis

The structure was analyzed by the curve fitting of PDF data simulated using the PDFfit2 program.^39^ Since the structure of magadiite was unknown, first, the initial structure model was prepared from the octosilicate structure^25^,^26^ to correspond to other data such as compositions, and NMR spectra and infrared spectra were investigated. The atomic coordinates of the structure model were moved to fit the experimental PDF data using a code running the real-space reverse Monte Carlo simulation^37^,^40^ implemented in the PDFfit2 program^39^ under bond length constraints (to retain SiO_4_ tetrahedra by keeping the Si–O bond length in the range of 1.45–1.75 Å and the O–O distance in the range of 2.50–2.80 Å) using the PDFfit2 program as the fitting program. For the structure model reaching *R*_w_ < 0.35, the symmetry of the structure was analyzed and then further refinements were carried out under the symmetry constraints. The initial symmetry used for the analysis was the *P*2_1_ space group, which was based on the literature where the symmetry of the Na-magadiite was considered to be monoclinic.^8^ However, during further investigations, we found that the symmetry of the local structure might correspond to the *P*1 space group to reach a better fit.

After refining the framework structure (in the *P*1 space group), we determined the unit cell of the whole crystal structure using the synchrotron XRD pattern with the structure model having the framework structure obtained by the PDF analysis. Rietveld analysis was carried out using the GSASII program.^41^ Note that since we did not assume stacking disorders in the model for the XRD analysis and also occupancies of the Na sites and water molecule sites were not clear enough (due to the disorders), the higher symmetry (*P*2_1_) might still be reasonable. However, because the samples were not highly crystalline, further detailed discussion of the symmetry of the crystal structure is future work and here we want to focus on the local-to-middle range structure, that is, the silicate framework structure.

Considering the reasonable fit of the PDF data of H-magadiite, the *P*2_1_ symmetry of the structure is reasonable at least in the local structure, though the symmetry at the crystal level might be different (but both H-magadiite and Na-magadiite may contain disorders such as stacking disorders, which have kept the structure unknown, and thus, our approach to determine the local symmetry is reasonable). The crystals of magadiites were found to contain stacking disorders as expected from the layered structure and furthermore we found zeolitic micropores in the layers where Na ions exist with disorders. The composition can be described also as H_2_Si_10_O_22_·1.44[Na(H_2_O)_4_], and the water molecules coordinated to Na ions might form Na(H_2_O)_6_ chains through edge-sharing connectivity. The size and volume of voids in the H-magadiite structure were analyzed by calculations using the Olex2 program.^42^

### Adsorption tests

Adsorption experiments of benzoic acid onto H-magadiite or H-octosilicate were conducted using acetonitrile solution as follows. The layered silicate (350 mg) was dispersed in a solution of acetonitrile (20 mL) containing benzoic acid or a mixed solution of acetonitrile containing toluene, benzyl alcohol, benzaldehyde and benzoic acid in a glass vessel. The vessel was sealed with a rubber septum and shaken for a day at room temperature. The supernatant was separated by filtration and quantitatively analyzed using a Shimadzu GC-2010 gas chromatograph with a flame ionization detector (GC-FID).

### Photocatalytic reactions

Pt-loaded P25 (100 mg), with or without layered silicates (200 mg), was dispersed in a solution of acetonitrile (5 mL) containing toluene (2 mmol) in a Pyrex glass tube (34 mL). After bubbling with O_2_ gas, the glass tube was sealed with a rubber septum and irradiated with a solar simulator (San-Ei Electric, *λ* > 300 nm, 1000 Wm^–2^) under stirring. After the irradiation, the headspace gas was withdrawn with a gas-tight syringe and quantified using a Shimadzu GC-8A gas chromatograph with a thermal conductivity detector. Then, the supernatant was separated by filtration and quantitatively analyzed by GC-FID. The solid, on the other hand, was washed with 100 mL of aqueous ethanol (1 : 1 v/v) and the eluate was quantitatively analyzed by GC-FID.

## Conflicts of interest

There are no conflicts to declare.

## Supplementary Material

Supplementary information
