# Supplementary material for: Zeolitic intralayer microchannels of magadiite, a natural layered silicate, to boost green organic synthesis
Source: Chem Sci. 2018 Nov 2;9(46):8637–43. doi: 10.1039/c8sc03712d (PMC6335629; doi:10.1039/c8sc03712d)
Supplement: Supplementary file 1 [file SC-009-C8SC03712D-s001.pdf]

Electronic Supplementary Material (ESI) for Chemical Science.
This journal is © The Royal Society of Chemistry 2018

Electronic Supplementary Information (ESI) for:

Zeolitic Intralayer Microchannels of Magadiite, a Natural Layered Silicate, to Boost Green Organic Synthesis

Yusuke Ide,*[a†] Satoshi Tominaka,*[a†] Hiroyuki Kono,[b] Rahul Ram,[a,c] Akihiko Machida[d] and Nao Tsunoji[e]

[a] International Center for Materials Nanoarchitectonics (MANA), National Institute for Materials Science, 1-1 Namiki, Tsukuba, Ibaraki 305-0044, Japan. E-mail: IDE.Yusuke@nims.go.jp (YI); TOMINAKA.Satoshi@nims.go.jp (ST)

[b] Department of Earth Sciences, Waseda University, 1-6-1 Nishiwaseda, Shinjuku-ku, Tokyo 165-8050, Japan.

[c] Center for Education, CSIR-Central Electrochemical Research Institute, Karaikudi, Tamil Nadu, India 630006.

[d] Synchrotron Radiation Research Center, National Institutes for Quantum and Radiological Science and Technology, 1-1-1, Kouto, Sayo-cho, Sayo-gun, Hyogo 679-5148, Japan.

[e] Graduate School of Engineering, Department of Applied Chemistry, Hiroshima University, 1-4-1 Kagamiyama, Higashi-Hiroshima 739-8527, Japan.

† These authors contributed equally.

Contents

1. Detailed method

2. Supplementary data (Table S1, Figure S1-S11)
   - Table S1: Elemental analysis
   - Fig. S1, S2: Illustration of Na-magadiite structure
   - Fig. S3: TGA
   - Fig. S4, S5: Solid-state NMR spectra
   - Fig. S6: FTIR
   - Fig. S7: SEM of Na-magadiite
   - Fig. S8: Additional PDFs and XRD patterns
   - Fig. S9: N_2 adsorption/desorption isotherms
   - Fig. S10: Solvent accessible pores
   - Fig. S11: Photocatalytic test data

3. References

4. Structure information

Detailed Methods

Preparation of materials. Na-magadiite was purchased from Nippon Chemical Industrial and used as received. The quality of the sample was confirmed by X-ray diffractometry (XRD), which shows a typical pattern for Na-magadiite (**Fig. 1, Fig. S1 and S2**)[1-3]. We determined the composition by the inductively coupled plasma optical emission spectroscopy (ICP-OES) using Hitachi HT ICP-OES SPS3520UV-DD for Na and Si ions (**Table S1**) and thermogravimetric analysis (TGA) using Hitachi HT-Seiko Instrument Exter 6300 for H_2O (**Fig. S3**). The Na/Si molar ratio is in good agreement with those reported in the literature[1,4]. Na-magadiite exhibits endothermic weight losses: (*i*) ~13 wt% loss from room temperature to 160°C, and (*ii*) gradual 1.9 wt% loss up to 700°C. The former appears to consist of two steps and is consistent with previous reports[3-6].

The protonation of Na-magadiite and Na-octosilicate was carried out based on previous reports[7,8]. The composition analysis confirms the almost complete removal of Na ions from Na-magadiite (Table S1). The TGA profile exhibits a typical one for H-magadiite[5]; weight losses: (*i*) ~0.6 wt% loss from room temperature to 200°C, (*ii*) ~3.1 wt% endothermic loss from 200°C to 440°C and (*iii*) gradual ~1.3 wt% endothermic loss up to 1000°C (Fig. S3).

The composition of Na-magadiite was thus determined to be $Na_{1.44}Si_{10}O_{22}\cdot 5.7H_2O$. This composition is identical to the structure model used for PDF fitting ($Na_{1.44}Si_{10}O_{22}\cdot 3H_2O$), where the amount of H_2O may have error because it simulates the tiny peak around 2.15 Å mostly. The sodium aqua complex is considered to be a coordination polymer of $[Na(H_2O)_{3.0}(OH)_{1.0}]_m$. Considering the following NMR results (**Fig. S4**), the structure contains silanol groups, and the composition for the unit cell should be $H_4Si_{10}O_{22}\cdot 1.44[Na(H_2O)_{4.0}]$. The composition of the protonated magadiite was determined likewise, $H_{3.6}Si_{10}O_{22}\cdot nH_2O$ ($n < 0.3$).

Materials characterizations. Nitrogen and water vapor adsorption/desorption isotherms were measured at 77 K and 298 K, respectively, on a BELSORP-max instrument (MicrotracBEL). Prior to the measurements, H-magadiite and H-octosilicate were outgassed at 573 K for 24 h and 393 K for 3 h, respectively. Synchrotron powder XRD patterns were collected at BL5S2 (λ = 1.29945 Å), AichiSR, at 25°C. Solid-state ^{29}Si NMR spectrum was recorded at 119.17 MHz on a Varian 600PS solid NMR spectrometer using a 6-mm diameter zirconia rotor. The magic-angle spinning (MAS) spectrum of Na-magadiite indicates the Q^3/Q^4 peak integral ratio of 38.8/61.2, whose Q^3 Si was confirmed to be protonated through the comparison with cross-polarization (CP) spectrum where Q^3/Q^4 increased obviously (Fig. S4). Likewise, MAS NMR spectrum of H-magadiite indicates the Q^3/Q^4 ratio of 28.5/71.5. The Q^3/Q^4 ratios for Na-magadiite and H-magadiite are within reported values[1,2,9] and in good agreement with the reported values[7,9], respectively. The discrepancy in the Q^3/Q^4 ratio between Na-magadiite and H-magadiite probably originates from partial condensation reactions between hydroxyl groups during the protonation of Na-magadiite[1]. Likewise, solid-state ^{1}H NMR spectra of Na-magadiite and H-magadiite were recorded (**Fig. S5**).

The infrared spectra were measured using Thermoscientific Nicolet 4700 spectrometer in the transmission configuration for the samples pelletized with KBr powder. **Fig. S6** shows IR spectra of Na-magadiite and H-magadiite. The spectra were normalized on the basis of the peak assignable to Si-O-Si asymmetric stretching (1000–1130 cm^{-1}). Na-magadiite exhibits a typical spectrum reported in the literatures[1,2,10,11]. The spectrum for Na-magadiite has a large H-O-H bending band (1630 and 1660 cm^{-1}) than that for H-magadiite, and only the former has a sharp O-H stretching band at 3660 cm^{-1}. These features can be explained by that Na-magadiite adsorbs a larger amount of water than H-magadiite as a result of interaction between Na^+ ions and water molecules.[5,6,11].

The crystal morphology of Na-magadiite was observed using a Hitachi S-4800 scanning electron microscope (SEM). Na-magadiite was composed of platy particles that form rosette (cabbage)-like spherical aggregates with several μm diameter (**Fig. S7**), a well-known characteristic of Na-magadiite[1].

X-ray PDF measurements. X-ray total scattering data for obtaining pair distribution functions (PDFs) were collected on a Rigaku Rapid-S curved imaging plate detector with Ag Kα radiation (λ = 0.556 Å) for screening structure models initially. The samples were sealed in Cole-Parmer polyimide capillaries (inner diameter: 1.0 mm). These corrected intensities were normalized by the Faber-Ziman type scattering form factors calculated using atomic scattering factors to obtain structure functions, $S(Q)$. The $S(Q)$ (Q_{max} = 21.0 $Å^{-1}$) was treated with a revised Lorch function (Δ = 1.00)[12], and then converted into reduced PDF, $G(r)$, where r is the interatomic distance.

High-resolution PDF data was obtained using synchrotron irradiation at BL22XU (λ = 0.1774 Å) and BL08W (λ = 0.1076 Å) in SPring-8 with a Perkin Elmer flat panel detector (XRD1621). The former results in PDFs with reasonably good spatial resolution, Q_{max} = 25.5 $Å^{-1}$, and also good angular resolution enabling analysis of long-range region in real space. The latter results in PDFs with high spatial resolution, Q_{max} = 33.0 $Å^{-1}$, but with little angular resolution.

Details of structure analysis.

The structure was analyzed by the curve fitting of PDF data simulated using the PDFfit2 program[13]. Since the structure of magadiite was unknown, hundreds of structure models which can also correspond to other data such as compositions, NMR spectra and infrared spectra were investigated. For some of those structure models, atomic coordinates were moved to fit the experimental PDF data using a code running the real-space Reverse Monte Caro simulation[14] implemented in the PDFfit2 program[13] under bond length restraints (to retain SiO_4 tetrahedra by keeping Si-O bond length in the

range of 1.45–1.75 Å and O-O distance in 2.50–2.80 Å) using the PDFfit2 program as the fitting program. Hundreds of structure models were screened and investigated as initial models, and then reasonable models were gradually selected.

For the structure model reaching $R_w < 0.35$, symmetry of the structure was analyzed and then further refinements were carried out under the symmetry constraints. The initial symmetry used for the analysis was $P2_1$ space group, which was based on literatures where the symmetry of the Na-magadiite considered to be monoclinic[1]. However, during further investigations, we found the symmetry of local structure should be in the $P\bar{1}$ space group to reach better fit. Considering the reasonable fit of PDF data and XRD data of H-magadiite, the symmetry of the structure is reasonable at least in the local structure, though symmetry in crystal level might be different (but both H-magadiite and Na-magadiite contain disorders such as turbostratic stacking disorder and stacking faults, which have been keeping the structure unknown, and thus, our approach to determine local symmetry is reasonable). The crystals of magadiites were found to contain stacking disorders as expected from the layered structure and furthermore we found zeolitic micropores in the layers where Na ions exist with disorders. The composition can be described also as $H_2Si_{10}O_{22}\cdot 1.44[Na(H_2O)_4]$, and the water molecules coordinated to Na ions might be formed $Na(H_2O)_6$ chains through edge-sharing connectivity. The size and volume of voids in the H-magadiite structure were analyzed by the calculations using the Olex2 program[15].

Quantum chemical calculations. In order to acquire better understanding of the adsorption of the benzoic acid molecules in the microchannels of magadiite, quantum chemical calculations were performed. The initial structure model was formed by adjusting the experimentally determined H-magadiite structure to have a 2×1×1 supercell, which can contain one benzoic acid molecule. The O coordinates and benzoic acid molecule was optimized by the density functional theory (DFT) using

the CASTEP program with the generalized gradient approximation (GGA) and the Perdew, Burke and Ernzerhof (PBE) exchange-correlation functional. The ultra-soft pseudopotentials1 and a 160-eV energy cut-off were used.

Supplementary Table and Figures

Table S1. Compositions of Na-magadiite and H-magadiite.

	Na (wt%)	Si (wt%)
Na-magadiite	4.2	35.3
H-magadiite	<0.01	37.4

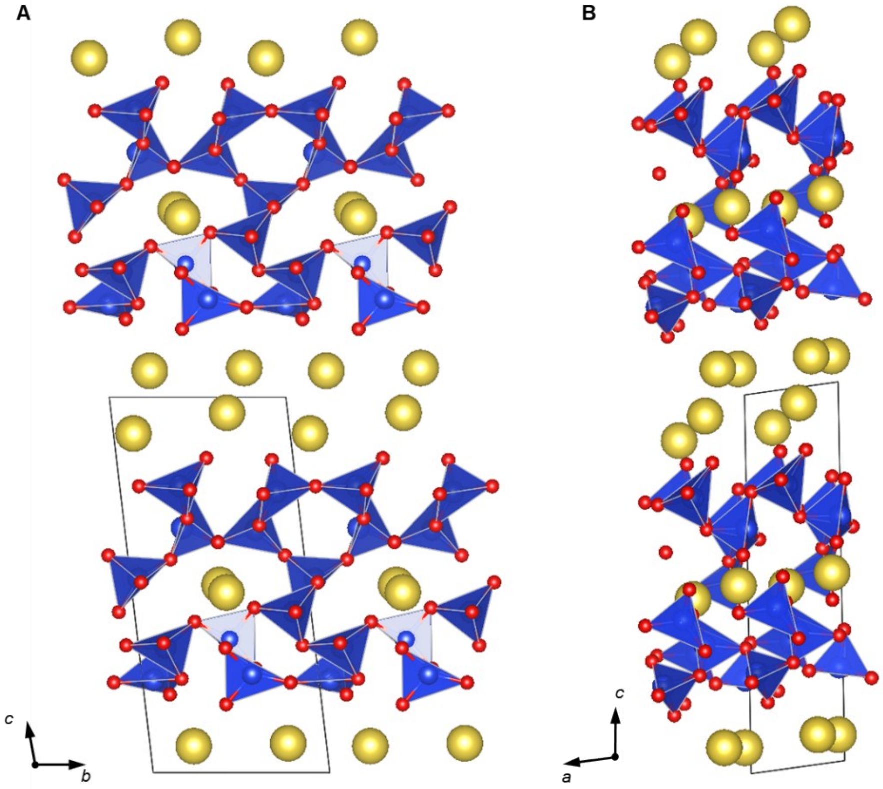

Fig. S1. Structure model of Na-magadiite obtained by the XRD analysis. Colour coding: blue = Si, red = O, yellow = Na. This structure was visualized using the VESTA program.

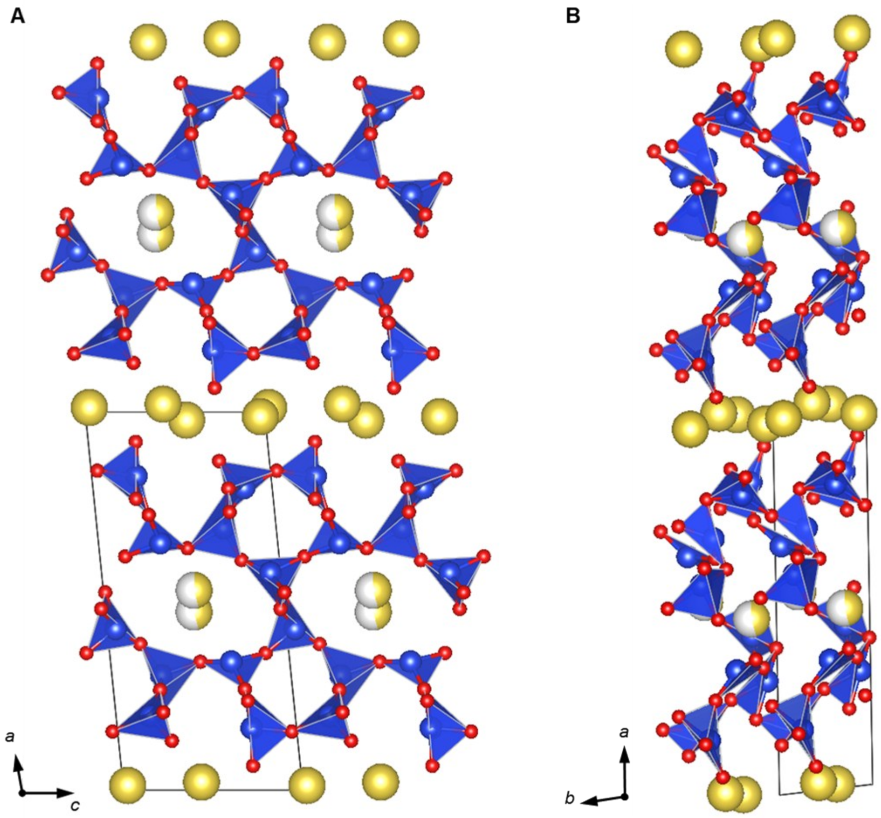

Fig. S2. Structure model of Na-magadiite obtained by the PDF analysis. Colour coding: blue = Si, red = O, yellow = Na. The yellow-white balls are Na with a low occupancy. This structure was visualized using the VESTA program.

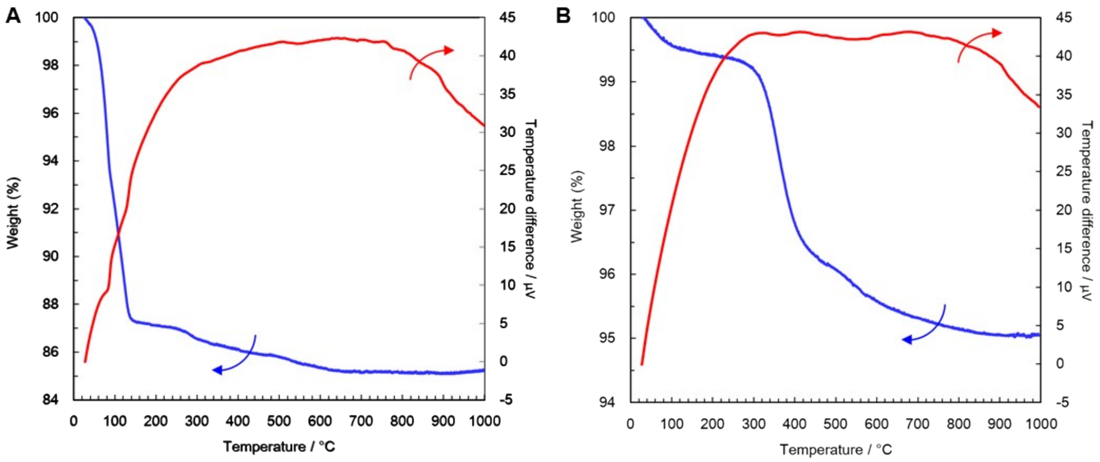

Fig. S3. Simultaneous thermogravimetric analysis and differential thermal analysis (TG/DTA). (A) Na-magadiite. **(B)** H-magadiite measured in air. Scan rates are 5 °C/min for Na-magadiite and 10 °C/min for H-magadiite.

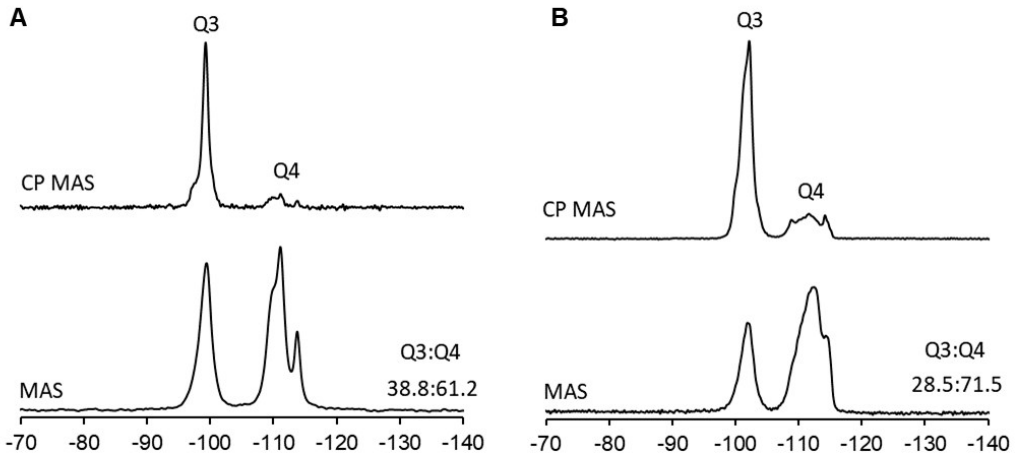

Fig. S4. Solid-state ^{29}Si NMR spectra of (A) Na-magadiite and (B) H-magadiite.

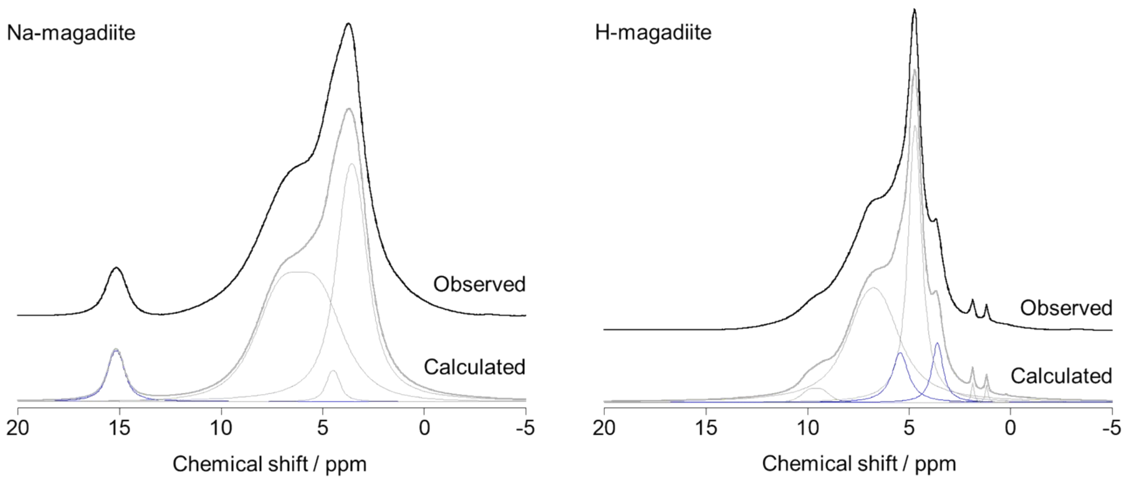

Fig. S5. Solid-state ^{1}H NMR spectra of Na-magadiite and H-magadiite. Calculated spectra were obtained by assuming the four and eight components, respectively. A peak at 15.2 ppm for Na-magadiite is assigned to the interlayer silanol group (SiOH···$^-$OSi).[16] Peaks at 5.4 and 3.6 ppm for H-magadiite are assigned to the interlayer silanol groups (strongly and weakly interacted SiOH, respectively).[17] Other peaks are assignable to H_2O molecules.[16,17] The integral ratio of peaks associated with the SiOH groups for H-magadiite to that for Na-magadiite is 2.3.

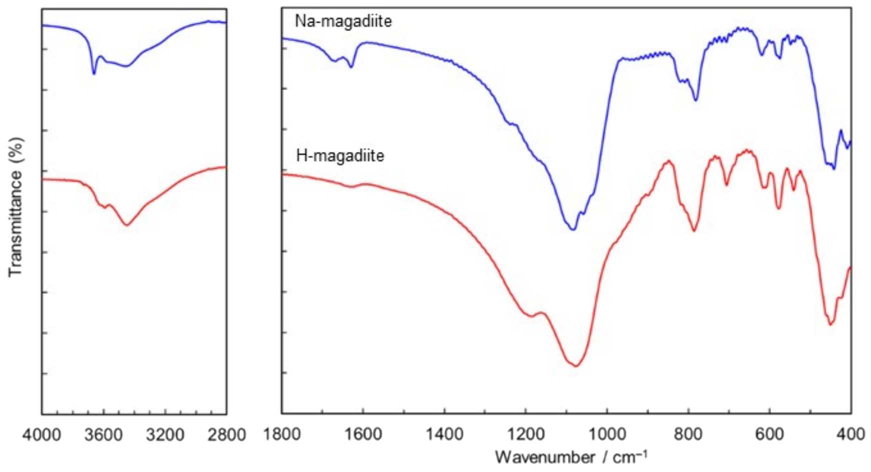

Fig. S6. FT-IR spectra of Na-magadiite and H-magadiite. The spectra were normalized on the basis of the absorption of Si-O-Si asymmetric stretching (1000–1130 cm^{-1}) and were shown with shift for clarity.

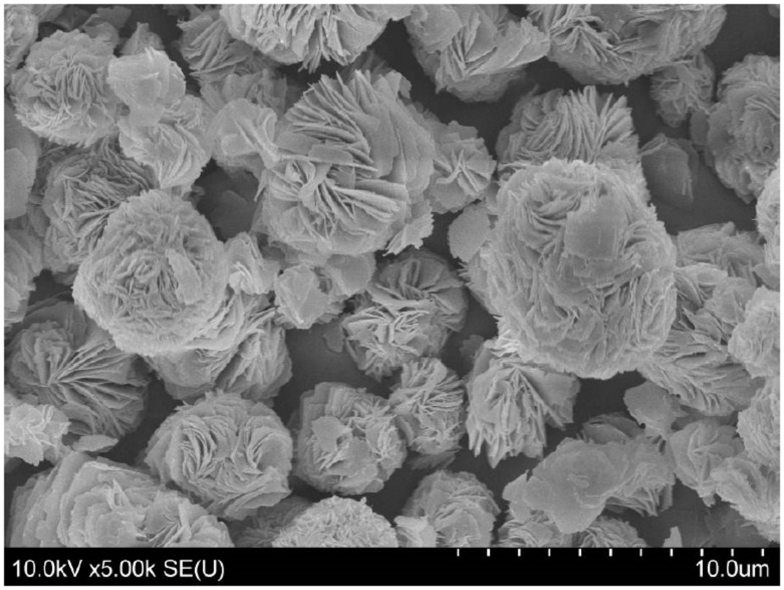

Fig. S7. SEM image of Na-magadiite.

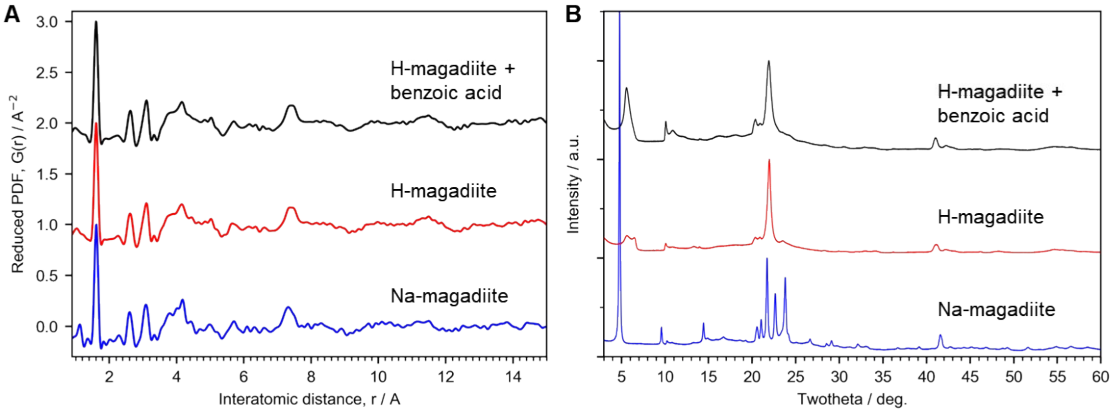

Fig. S8. Comparison of X-ray data of magadiite samples: Na-magadiite, H-magadiite before and after benzoic acid uptake. (A) High special resolution PDF data (Q_{max} = 33.0 Å^{-1}) collected at BL08W (λ = 0.1076 Å), SPring-8. **(B)** Synchrotron PXRD patterns collected at BL5S2 (λ = 1.29945 Å), AichiSR.

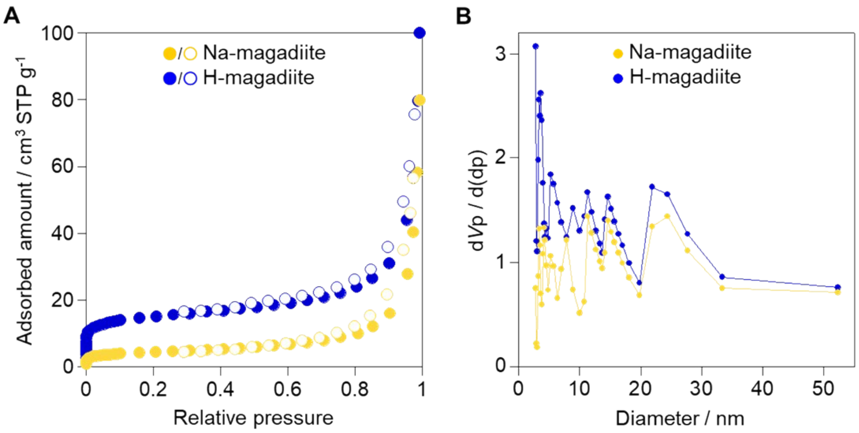

Fig. S9. Mesoporosity of Na-magadiite and H-magadiite. (A) N_2 adsorption (filled)/desorption (open) isotherms and **(B)** BJH pore size distribution of Na-magadiite and H-magadiite. Na-magadiite (mesopore volume of 0.0055 $cm^3\ g^{-1}$) and H-magadiite (0.067 $cm^3\ g^{-1}$) have few meso-porosity due to the low adsorption value at high relative pressure and random pore size distribution.

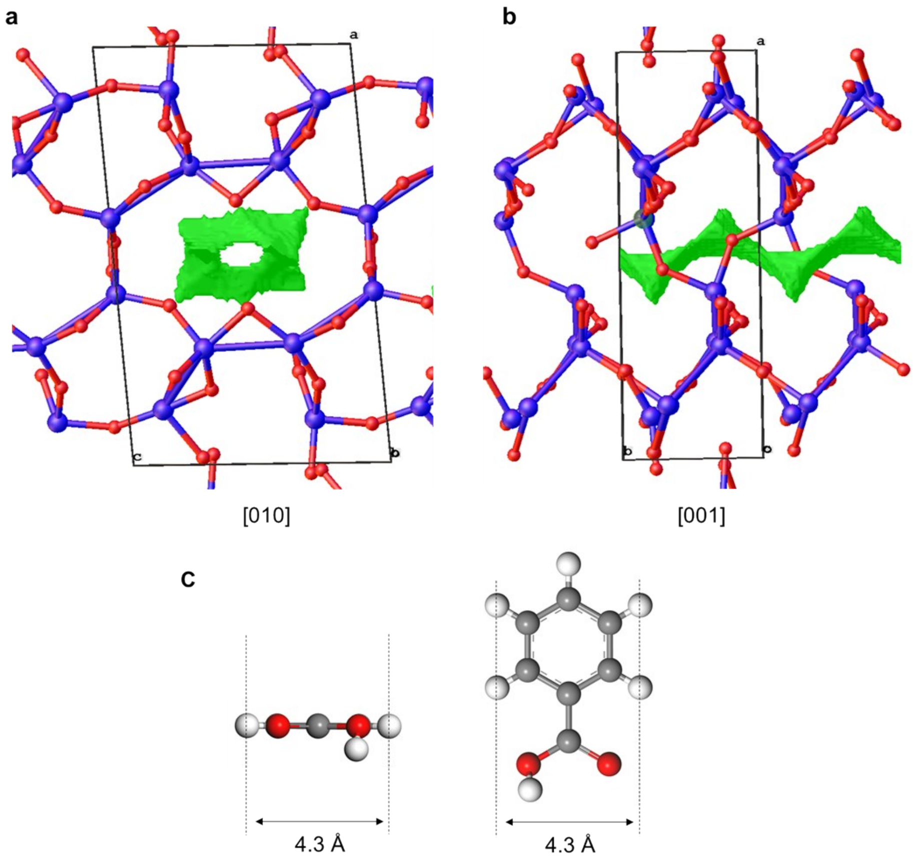

Fig. S10. Framework and voids in H-magadiite. (a) Parallel view along the *b* axis. **(b)** Parallel view along the *c* axis. The green regions illustrated in the panel ‘a’ and ‘b’ show the void calculated using the Olex2 program (resolution = 0.1 Å, and distance = 0.0 Å). The voids are solvent accessible pores along the *b* axis, and the diagonals of the cross-section of the void are ca. 5.5 Å (longitudinal) and ca. 2.1 Å. The structure occupies 317.06 $Å^3$ (88.3% of the unit cell), and the void does 42.83 $Å^3$ (11.7%, 0.0 Å away from the surface) or 22.79 $Å^3$ (6.2%, 0.2 Å away from the surface). **(c)** Size of benzoic acid molecule, whose structure was optimized through the quantum chemical simulation using CASTEP program with the generalized gradient approximation (GGA) and the Perdew, Burke and Ernzerhof (PBE) exchange-correlation functional. The ultra-soft pseudopotentials1 and a 340-eV energy cut-off were used.

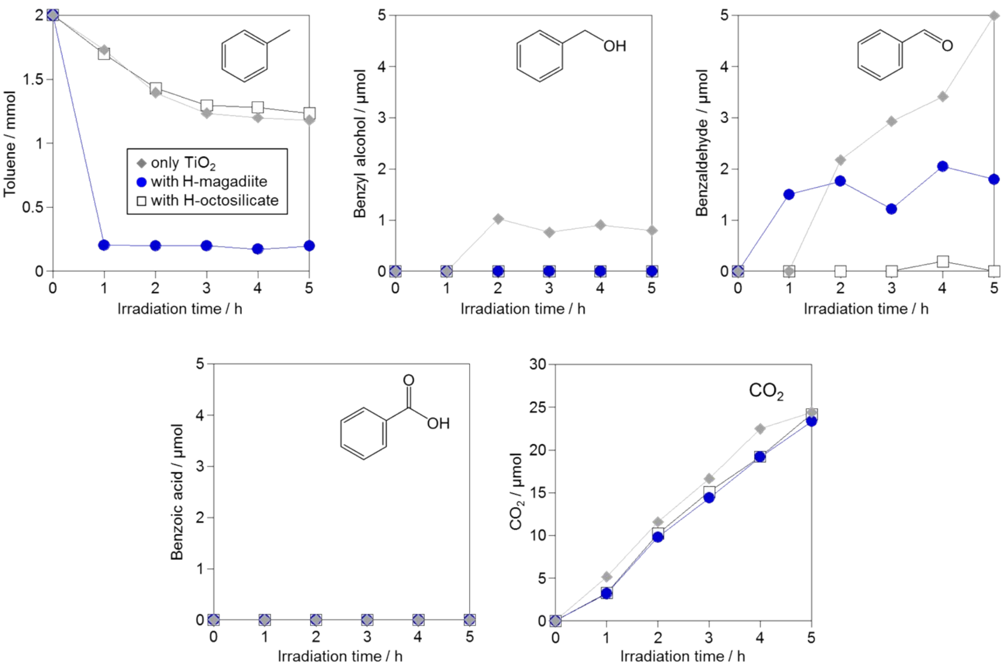

Fig. S11. Time course of consumption of toluene and formation of oxidized products during the photocatalytic oxidation of toluene on TiO_2 with or without adsorbents.

References

1. Garcés, J. M.; Rocke, S. C.; Crowder, C. E.; Hasha, D. L. Hypothetical structures of magadiite and sodium octosilicate and structural relationships between the layered alkali metal silicates and the mordenite- and pentasil-group zeolites. *Clays Clay Miner.* **1988**, *36*, 409-418.
2. Scholzen, G.; Beneke, K.; Lagaly, G. Diversity of magadiite. *Z. Anorg. Allg. Chem.* **1991**, *597*, 183-196.
3. Sassi, M.; Miehé-Brendlé, J.; Patarin, J.; Bengueddach, A. Na-magadiite prepared in a water/alcohol medium: synthesis, characterization and use as a host material to prepare alkyltrimethylammonium- and Si-pillared derivates. *Clay Miner.* **2005**, *40*, 369-378.
4. Lagaly, G.; Beneke, K. Magadiite and H-magadiite: I. Sodium magadiite and some of its derivative. *Am. Miner.* **1975**, *60*, 642-649.
5. Rojo, J. M.; Ruiz-Hitzky, E. J. Sanz, Proton-sodium exchange in magadiite. Spectroscopic study (NMR, IR) of the evolution of interlayer OH groups, *Inorg. Chem.* **1988**, *27*, 2785-2790.
6. Eypert-Blaison, C.; Sauzéat, E.; Pelletier, M.; Michot, L. J.; Villiéras, F.; Humbert, B. Hydration mechanisms and swelling behavior of Na-magadiite, *Chem. Mater.* **2001**, *13*, 1480-1486 (2001).
7. Asakura, Y.; Hosaka, N.; Osada, S.; Terasawa, T.; Shimojima, A.; Kuroda, K. Interlayer condensation of protonated layered silicate magadiite through refluxing in *N*-methylformamide. *Bull. Chem. Soc. Jpn.* **2015**, *88*, 1241-1249.
8. Borowski, M.; Kovalev, O.; Gies, H. Structural characterization of the hydrous layer silicate Na-RUB-18, $Na_8Si_{32}O_{64}(OH)_8 \cdot 32H_2O$ and derivatives with XPD-, NPD-, and SS NMR experiments. *Microporous Mesoporous Mater.* **2008**, *107*, 71-80.

9. Pinnavaia, T. J.; Johnson, I. D. A ^{29}Si MAS NMR study of tetrahedral site distribution in the layered silicic acid H^+-magadiite ($H_2Si_{14}O_{29}\cdot nH_2O$) and in Na^+-magadiite ($Na_2Si_{14}O_{29}\cdot nH_2O$). *J. Solid State Chem.* **1986**, *63*, 118-121.

10. Huang, Y.; Jiang, Z.; Schwieger, W. Vibrational spectroscopic studies of layered silicates. *Chem. Mater.* **1999**, *11*, 1210-1217.

11. Eypert-Blaison, C.; Humbert, B.; Michot, L. J.; Pelletier, M.; Sauzéat, E.; Villiéras, F. Structural role of hydration water in Na- and H-magadiite: A spectroscopic study, *Chem. Mater.* **2001**, *13*, 4439-4446.

12. Soper, A. K.; Barney, E. R. Extracting the pair distribution function from white-beam X-ray total scattering data. *J. Appl. Crystallogr.* **2011**, *44*, 714-726.

13. Farrow, C. L.; Juhas, P.; Liu, J. W.; Bryndin, D.; Bozin, E. S.; Bloch, J.; Proffen, T.; Billinge S. J. L. PDFfit2 and PDFgui: Computer programs for studying nanostructure in crystals. *J. Phys.-Condens. Mat.* **2007***, 19*, art. no. 335219.

14. Tominaka, S.; Kawakami, K.; Fukushima, M.; Miyazaki, A. Physical stabilization of pharmaceutical glasses based on hydrogen bond reorganization under sub-T_g temperature. *Mol. Pharmaceut.* **2017***, 14*, 264-273.

15. Dolomanov, O. V.; Bourhis, L. J.; Gildea, R. J.; Howard, J. A. K.; Puschmann, H. OLEX2: A complete structure solution, refinement and analysis program. *J. Appl. Crystallogr.* **2009**, *42*, 339-341.

16. Almond, G. A.; Harris, R. K.; Graham, P. A study of the layered alkali metal silicate, magadiite, by one- and two-dimensional ^1H and ^{29}Si NMR Spectroscopy. *J. Chem. Soc., Chem. Commun.* **1994**, 851-852.

17. Komori, Y.; Miyoshi, M.; Hayashi, S.; Sugahara, Y.; Kuroda, K. Characterization of silanol groups in protonated magadiite by ^{1}H and ^{2}H solid-state nuclear magnetic resonance. *Clays Clay Miner.* **2000**, *48*, 632-637.

Structure information

We solved the structures in the unit cells shown below. Then, for the clear comparison, we transform the cell of the H-magadiite to have the similar beta value as shown in the main text.

(1) Na-mag, refined by XRD

```
#######################################################################

_cell_length_a  15.748821
_cell_length_b  3.929695
_cell_length_c  7.365532
_cell_angle_alpha  96.3874
_cell_angle_beta   95.9183
_cell_angle_gamma  96.177
_cell_volume  447.313
_symmetry_cell_setting  triclinic
_symmetry_space_group_name_H-M  P1
loop_
    _space_group_symop_id
    _space_group_symop_operation_xyz
     1  x,y,z

# ATOMIC COORDINATES AND DISPLACEMENT PARAMETERS
loop_
   _atom_site_label
   _atom_site_type_symbol
   _atom_site_fract_x
   _atom_site_fract_y
   _atom_site_fract_z
   _atom_site_occupancy
   _atom_site_adp_type
   _atom_site_U_iso_or_equiv
   _atom_site_symmetry_multiplicity
Si1   Si   0.647(4)   1.005(19)   0.742(8)    1.000    Uiso 0.226      1
Si2   Si   0.764(4)   0.680(18)   0.340(10)   1.000    Uiso 0.226108   1
Si3   Si   0.429(4)   0.694(17)   0.906(10)   1.000    Uiso 0.226108   1
Si4   Si   0.355(5)   0.472(17)   0.520(11)   1.000    Uiso 0.226108   1
Si5   Si   0.248(5)   1.042(20)   0.062(12)   1.000    Uiso 0.226108   1
O1    O    0.345(5)   0.981(18)   0.145(11)   1.000    Uiso 0.347      1
O2    O    0.753(4)   1.081(18)   0.395(11)   1.000    Uiso 0.347236   1
O3    O    0.568(5)   1.198(18)   0.239(9)    1.000    Uiso 0.347236   1
O4    O    0.833(4)   0.356(20)   0.879(13)   1.000    Uiso 0.347236   1
O5    O    0.428(4)   1.101(17)   0.912(10)   1.000    Uiso 0.347236   1
O6    O    0.763(6)   0.627(18)   0.109(9)    1.000    Uiso 0.347236   1
O7    O    0.402(5)   0.529(20)   0.335(10)   1.000    Uiso 0.347236   1
O8    O    0.677(4)   0.446(17)   0.360(12)   1.000    Uiso 0.347236   1
```

O9	O	0.250(5)	0.363(20)	0.223(11)	1.000	Uiso 0.347236	1
O10	O	0.339(4)	0.469(17)	-0.069(11)	1.000	Uiso 0.347236	1
O11	O	0.182(5)	0.809(19)	0.458(13)	1.000	Uiso 0.347236	1
Na1	Na	0.48000	0.58818	0.53299	1.000	Uiso 0.300	1
Na2	Na	-0.09638	0.70595	0.11257	2.07(13)	Uiso 0.340	1
Na3	Na	0.07073	0.06213	0.24665	2.57(10)	Uiso 0.297	1
Si6	Si	0.334(5)	0.556(18)	0.152(11)	1.000	Uiso 0.226108	1
Si7	Si	0.263(4)	0.918(18)	0.624(12)	1.000	Uiso 0.226108	1
Si8	Si	0.516(4)	1.135(19)	1.045(8)	1.000	Uiso 0.226108	1
Si9	Si	0.652(4)	1.010(17)	0.325(9)	1.000	Uiso 0.226108	1
Si10	Si	0.748(4)	0.568(18)	0.883(10)	1.000	Uiso 0.226108	1
O12	O	0.659(5)	0.428(19)	0.764(10)	1.000	Uiso 0.347236	1
O13	O	0.332(4)	1.036(16)	0.491(11)	1.000	Uiso 0.347236	1
O14	O	0.439(5)	0.599(18)	0.682(10)	1.000	Uiso 0.347236	1
O15	O	0.208(6)	0.698(24)	0.146(13)	1.000	Uiso 0.347236	1
O16	O	0.507(4)	0.625(18)	0.057(9)	1.000	Uiso 0.347236	1
O17	O	0.240(5)	0.976(19)	0.837(12)	1.000	Uiso 0.347236	1
O18	O	0.614(4)	0.870(18)	0.519(8)	1.000	Uiso 0.347236	1
O19	O	0.277(4)	0.530(18)	0.648(11)	1.000	Uiso 0.347236	1
O20	O	0.742(4)	0.946(18)	0.808(10)	1.000	Uiso 0.347236	1
O21	O	0.579(4)	1.046(20)	0.890(9)	1.000	Uiso 0.347236	1
O22	O	0.838(6)	0.628(22)	0.509(12)	1.000	Uiso 0.347236	1
Na4	Na	0.50000	0.10000	0.50000	1.000	Uiso 0.300	1
Na5	Na	-0.04108	1.42472	0.65647	2.30(13)	Uiso 0.334	1
Na6	Na	0.07817	1.28148	0.78369	2.74(11)	Uiso 0.314	1

(2) Na-mag, refined by PDF fitting

```
##########################################################################
_symmetry_space_group_name_H-M  'P1'
_symmetry_Int_Tables_number     1
_symmetry_cell_setting          triclinic

_cell_length_a                  15.5996
_cell_length_b                  3.83664
_cell_length_c                  7.34442
_cell_angle_alpha               93.7639
_cell_angle_beta                95.4159
_cell_angle_gamma               95.2027

loop_
  _atom_site_label
  _atom_site_type_symbol
  _atom_site_fract_x
  _atom_site_fract_y
  _atom_site_fract_z
  _atom_site_U_iso_or_equiv
  _atom_site_adp_type
  _atom_site_occupancy
  Si1   Si   0.693175   0.661899   0.704406   0.018177 Uiso  1.0000
  Si2   Si   0.303214   0.111840   0.276525   0.018177 Uiso  1.0000
  Si3   Si   0.830529   0.244400   0.248790   0.018177 Uiso  1.0000
  Si4   Si   0.164281   0.739825   0.750757   0.018177 Uiso  1.0000
  Si5   Si   0.426759   0.261853   0.045479   0.018177 Uiso  1.0000
  Si6   Si   0.574979   0.799673   0.942358   0.018177 Uiso  1.0000
  Si7   Si   0.339429   0.433836   0.673751   0.018177 Uiso  1.0000
  Si8   Si   0.653809   0.938591   0.344674   0.018177 Uiso  1.0000
  Si9   Si   0.195581   0.788936   0.190820   0.018177 Uiso  1.0000
  Si10  Si   0.799429   0.342976   0.842453   0.018177 Uiso  1.0000
  O1    O    0.264991   0.497611   0.260553   0.007737 Uiso  1.0000
  O2    O    0.735935   0.031702   0.749073   0.007737 Uiso  1.0000
  O3    O    0.770258   0.578879   0.233834   0.007737 Uiso  1.0000
  O4    O    0.235872   0.062662   0.744219   0.007737 Uiso  1.0000
  O5    O    0.618052   0.762328   0.141541   0.007737 Uiso  1.0000
  O6    O    0.362770   0.228178   0.870811   0.007737 Uiso  1.0000
  O7    O    0.870590   0.463863   0.719441   0.007737 Uiso  1.0000
  O8    O    0.130241   0.966271   0.297398   0.007737 Uiso  1.0000
  O9    O    0.479465   0.626344   0.977584   0.007737 Uiso  1.0000
  O10   O    0.523045   0.163136   0.009479   0.007737 Uiso  1.0000
  O11   O    0.844788   0.306160   0.034712   0.007737 Uiso  1.0000
  O12   O    0.155073   0.782466   0.972049   0.007737 Uiso  1.0000
  O13   O    0.339351   0.148368   0.501111   0.007737 Uiso  1.0000
```

O14	O	0.639787	0.644838	0.493757	0.007737	Uiso	1.0000
O15	O	0.728030	0.222926	0.296058	0.007737	Uiso	1.0000
O16	O	0.267204	0.668042	0.733654	0.007737	Uiso	1.0000
O17	O	0.206140	0.235926	0.265807	0.007737	Uiso	1.0000
O18	O	0.797993	0.724803	0.755624	0.007737	Uiso	1.0000
O19	O	0.386603	0.007814	0.175723	0.007737	Uiso	1.0000
O20	O	0.608596	0.527548	0.788895	0.007737	Uiso	1.0000
O21	O	0.064237	0.603626	0.736119	0.007737	Uiso	1.0000
O22	O	0.921273	0.100503	0.265060	0.007737	Uiso	1.0000
Na1	Na	0.528633	0.766708	0.506142	0.006848	Uiso	0.4704
Na2	Na	0.471367	0.266708	0.493858	0.006848	Uiso	0.4704
Na3	Na	0.021234	0.578451	0.439113	0.006848	Uiso	1.0069
Na4	Na	0.978766	0.078451	0.560887	0.006848	Uiso	1.0069
Na5	Na	0.012134	0.386032	0.025603	0.006848	Uiso	1.0069
Na6	Na	0.987866	0.886032	0.974397	0.006848	Uiso	1.0069

(3) H-mag, refined by PDF fitting under the $P2_1$ symmetry constraint

```
#######################################################################
_symmetry_space_group_name_H-M  'P1'
_symmetry_Int_Tables_number     1
_symmetry_cell_setting          triclinic

_cell_length_a                  12.0636
_cell_length_b                  4.13469
_cell_length_c                  7.39298
_cell_angle_alpha               90
_cell_angle_beta                85.1961
_cell_angle_gamma               90

loop_
  _atom_site_label
  _atom_site_type_symbol
  _atom_site_fract_x
  _atom_site_fract_y
  _atom_site_fract_z
  _atom_site_U_iso_or_equiv
  _atom_site_adp_type
  _atom_site_occupancy
  Si1  Si   0.713682   0.817137   0.666770   0.005024 Uiso  1.0000
  Si2  Si   0.286318   0.317137   0.333230   0.005024 Uiso  1.0000
  Si3  Si   0.276720   0.284303   0.681978   0.005024 Uiso  1.0000
  Si4  Si   0.723280   0.784303   0.318022   0.005024 Uiso  1.0000
  Si5  Si   0.867705   0.169062   0.135781   0.005024 Uiso  1.0000
  Si6  Si   0.132295   0.669062   0.864219   0.005024 Uiso  1.0000
```

Si7	Si	0.408978	0.327489	0.002398	0.005024	Uiso	1.0000
Si8	Si	0.591022	0.827489	-0.002398	0.005024	Uiso	1.0000
Si9	Si	0.894279	0.292535	0.721079	0.005024	Uiso	1.0000
Si10	Si	0.105721	0.792535	0.278921	0.005024	Uiso	1.0000
O1	O	0.188456	0.518211	0.251557	0.019375	Uiso	1.0000
O2	O	0.811544	0.018211	0.748443	0.019375	Uiso	1.0000
O3	O	0.790211	0.486391	0.195466	0.019375	Uiso	1.0000
O4	O	0.209789	-0.013609	0.804534	0.019375	Uiso	1.0000
O5	O	0.781393	0.474369	0.676719	0.019375	Uiso	1.0000
O6	O	0.218607	0.974369	0.323281	0.019375	Uiso	1.0000
O7	O	0.178803	0.561316	0.668618	0.019375	Uiso	1.0000
O8	O	0.821197	0.061316	0.331382	0.019375	Uiso	1.0000
O9	O	0.619630	0.774131	0.200945	0.019375	Uiso	1.0000
O10	O	0.380370	0.274131	0.799055	0.019375	Uiso	1.0000
O11	O	0.665653	0.643583	0.849023	0.019375	Uiso	1.0000
O12	O	0.334347	0.143583	0.150977	0.019375	Uiso	1.0000
O13	O	0.023712	0.266970	0.685914	0.019375	Uiso	1.0000
O14	O	0.976288	0.766970	0.314086	0.019375	Uiso	1.0000
O15	O	0.907429	0.333282	0.940404	0.019375	Uiso	1.0000
O16	O	0.092571	0.833282	0.059596	0.019375	Uiso	1.0000
O17	O	0.370302	0.235230	0.496269	0.019375	Uiso	1.0000
O18	O	0.629698	0.735230	0.503731	0.019375	Uiso	1.0000
O19	O	0.465514	0.692626	0.008607	0.019375	Uiso	1.0000
O20	O	0.534486	0.192626	-0.008607	0.019375	Uiso	1.0000
O21	O	0.016697	0.777779	0.727444	0.019375	Uiso	1.0000
O22	O	0.983303	0.277779	0.272556	0.019375	Uiso	1.0000
